# 

**Published:** 1920-01-10

**Authors:** 


### Carpentry Clubs and Hospitals.

The decision of a number of residents in Ealing to form a Carpentry Club, with the object of making and providing articles required by local hospitals, might well be adopted in many other districts. It has also been suggested that the models, etc., made in school carpenter's shops might be in the nature of institutional appliances, and the scholars encouraged to give them where they were needed. Many articles in use are not of an intricate nature to make—*e.g.*, chart-boards, splints, etc.—whilst the skilled worker would find ample scope for his art in many ways, the ward lock and the screen being such examples.

### Gifts and Bequests.

WOLVERHAMPTON hospitals benefit under the will of Mr. John Scott, of Wolverhampton, as follows: Wolverhampton and South Staffordshire General Hospital, £5,000, for additions to be known as the "John Scott Wing"; £500 each to the Women's Hospital, the Eye Infirmary, and the Royal Orphanage. The Hon. Mrs. Magdalen Willesley left £100 each to the Hospital for Women, Soho Square, and the London Homœopathic Hospital.

THE Great Northern Central Hospital has received £260, the proceeds of an entertainment arranged by the Ingersoll Club, Regent Street.

MRS. E. AVINS, of Moseley, left the following amounts to Birmingham charities: Royal Orthopædic Hospital and Queen's Hospital, £2,000 each; Women's Hospital, Eye Hospital and Ear and Throat Hospital, £1,000 each; and £500 to Moseley Hall Convalescent Hospital. The ultimate residue of the estate is to be divided between the Royal Orthopædic Hospital and the Women's Hospital.

THE Chelsea Hospital for Women has received a bequest of £300 from the late Miss A. W. Harrison.

MR. HUGH VALLANCE, of Birmingham, has left £600 for charitable purposes.

### The College of Nursing (Limited by Guarantee.)

### An At-Home to be Attended by All

To celebrate the passing into law of the Nurses' Registration Act, Sir Arthur Stanley (Chairman) and the Council of the College of Nursing will be At Home at the Royal Automobile Club, Pall Mall, S.W., on Thursday, January 15, 1920, 4-6.30. The speakers will be the Right Hon. Viscount Sandhurst and the Right Hon. Christopher Addison, M.P., Minister of Health. Admission is by ticket only, which can be obtained from the Secretary of the College of Nursing, 7 Henrietta Street, W. 1. Invitation is extended to all members of the nursing profession, and others interested in the Nurses' Registration Act.

